# Patient-level proteomic network prediction by explainable artificial intelligence

**DOI:** 10.1038/s41698-022-00278-4

**Published:** 2022-06-07

**Authors:** Philipp Keyl, Michael Bockmayr, Daniel Heim, Gabriel Dernbach, Grégoire Montavon, Klaus-Robert Müller, Frederick Klauschen

**Affiliations:** 1 Institute of Pathology, Charité – Universitätsmedizin Berlin, Corporate Member of Freie Universität Berlin and Humboldt-Universität Berlin, Charitéplatz 1, 10117 Berlin, Germany; 2 Department of Pediatric Hematology and Oncology, University Medical Center Hamburg-Eppendorf, Martinistr. 52, 20246 Hamburg, Germany; 3 Mildred Scheel Cancer Career Center HaTriCS4, University Medical Center Hamburg-Eppendorf, Martinistr. 52, 20246 Hamburg, Germany; 4 BIFOLD – Berlin Institute for the Foundations of Learning and Data, Berlin, Germany; 5 Machine Learning Group, Technical University of Berlin, Marchstr. 23, 10587 Berlin, Germany; 6 Department of Artificial Intelligence, Korea University, Seoul, 136-713 South Korea; 7 Max-Planck-Institute for Informatics, Stuhlsatzenhausweg 4, 66123 Saarbrücken, Germany; 8 Institute of Pathology, Ludwig-Maximilians-University Munich, Thalkirchner Str. 36, 80337 München, Germany; 9 German Cancer Consortium (DKTK), German Cancer Research Center (DKFZ), Berlin Partner Site, Heidelberg, Germany; 10 German Cancer Consortium (DKTK), German Cancer Research Center (DKFZ), Munich Partner Site, Heidelberg, Germany

**Keywords:** Systems biology, Molecular medicine

## Abstract

Understanding the pathological properties of dysregulated protein networks in individual patients’ tumors is the basis for precision therapy. Functional experiments are commonly used, but cover only parts of the oncogenic signaling networks, whereas methods that reconstruct networks from omics data usually only predict average network features across tumors. Here, we show that the explainable AI method layer-wise relevance propagation (LRP) can infer protein interaction networks for individual patients from proteomic profiling data. LRP reconstructs average and individual interaction networks with an AUC of 0.99 and 0.93, respectively, and outperforms state-of-the-art network prediction methods for individual tumors. Using data from The Cancer Proteome Atlas, we identify known and potentially novel oncogenic network features, among which some are cancer-type specific and show only minor variation among patients, while others are present across certain tumor types but differ among individual patients. Our approach may therefore support predictive diagnostics in precision oncology by inferring “patient-level” oncogenic mechanisms.

## Introduction

Carcinogenesis involves a profound dysregulation of cellular control mechanisms that leads to excessive proliferation and evasion of apoptosis^1–3^. Proteins that participate in these dysregulated networks are potential pharmacological targets in precision oncology; however, the identification of the functionally relevant network modules is still subject to ongoing research^4^. Attempts are made trying to establish patient-derived functional models such as xenografts or organoids. However, the implementation of such models is technically challenging and often takes too long to be useful in routine diagnostics. Therefore, the ability to infer functional network information from proteomic profiling data even from routine diagnostic formalin-fixed tissue samples would entail great potential for diagnostics and therapy. Several methods for the inference of networks from cohort omics data have been proposed^5–9^. While many of these methods give insight into regulatory networks of homogeneous data as they are available from experimental model systems, the inference of regulatory networks for more complex, heterogeneous clinical data is a more demanding task. Here, the primary goal is not to find average population effects but to identify individual network characteristics that may be indicators of why some cancers metastasize or respond to treatment in a different way than others^10,11^. Precise information about the regulatory pathways in a tumor of an individual patient could help personalize treatment by specifically targeting dysregulated interactions and thus improve therapeutic efficacy^12^. LIONESS^13^ is a recently introduced method that can infer the regulatory interactions between genes or proteins for individual samples by linearly interpolating between two interaction networks reconstructed on the basis of cohort data. Here, we propose an approach that relies on a neural network model in combination with the explainable AI technology layer-wise relevance propagation (LRP)^14–18^ to predict regulatory networks from proteomic data for individual patients from a single sample^19^. Our approach is based on the assumption that if a neural network model is capable of reliably predicting the expression of a target protein based on the expression of a set of other source proteins, regulatory relationships exist between the source and the target proteins^20^. The explainable AI method LRP can then be used to infer the relevance of every source protein for the target prediction which can be interpreted as a measure for functional relationships between proteins. First approaches have shown that LRP can infer average interactions from multiple samples^21^. Here, we show that LRP can infer protein interaction networks even for individual patients and report differences and similarities of protein interaction networks across and within cancer types.

## Results

### Prediction of protein interaction across cancers

The reconstruction of protein interaction networks was based on proteomic data from The Cancer Proteome Atlas (TCPA)^22,23^.

We first chose the model hyperparameters (hidden layers, neurons per layer, learning rate, and number of training epochs) based on a 10-time repeated cross-validation. For every instance of validation, 50% of data was sampled as training data while the rest was held out as test data. The neural network model with three hidden layers, a learning rate of 0.03, and a neuron number of 10 times the input dimension showed the best performance (mean squared error: 0.48) on this task after training for 3600 epochs. It was therefore chosen for the final training and subsequent inference of protein interactions with LRP.

As a measure of the protein interaction strength, we computed the absolute undirected LRP scores *LRP*_*au*_. *LRP*_*au*_ is defined as mean of the two absolute LRP values *LRP*_*A*⇒*B*_ (relevance of protein A for the prediction of protein B) and *LRP*_*B*⇒*A*_ between two proteins A and B. We report median *LRP*_*au*_ scores across patient samples that are more robust than the mean against individual strong interactions to yield characteristic quantitative estimates of interaction strength in different cancer types. Among the 100 strongest median LRP interactions (out of 10,731), 56 interactions were described in the Reactome database (*p* = 1.1 ⋅ 10^−18^, hypergeometric test)^24,25^. In comparison, GENIE3, one of the state-of-the-art methods for network prediction, captured only 42 Reactome interactions with its highest 100 predictions (*p* = 3.8 ⋅ 10^−9^)^6^. The predicted interactions between unphosphorylated proteins and their phosphorylated variants showed the highest *LRP*_*au*_ interaction scores (median *LRP*_*au*_ between phosphorylated variants = 0.47, interquartile range (IQR) = 0.80; all other interactions: median *LRP*_*au*_ = 0.28, IQR = 0.31; *p* < 10^−16^, Mann–Whitney *U* test).

**Fig. 1:**
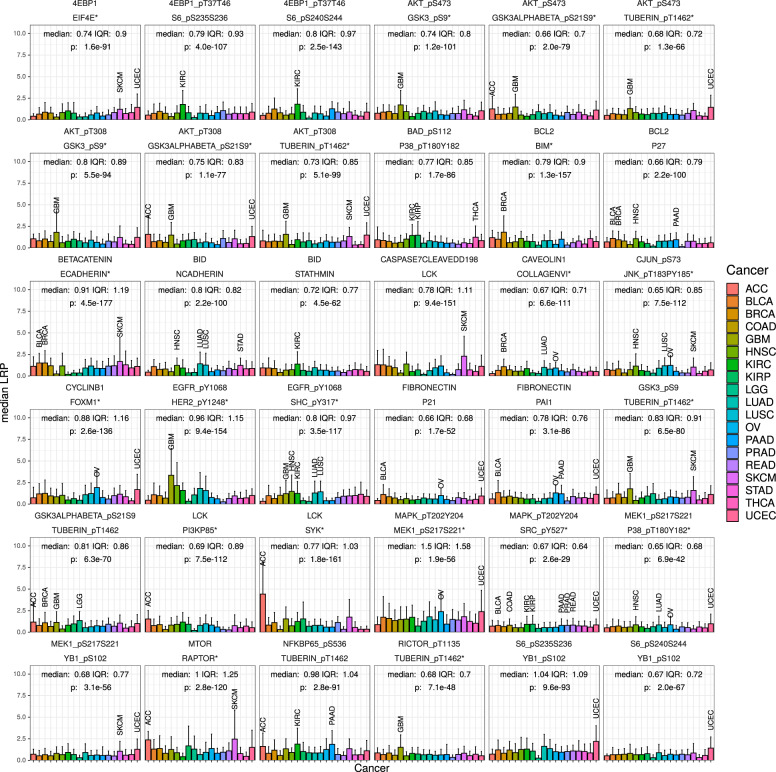
Protein interactions with top LRP scores in the TCPA data set. Each subplot shows the median *LRP*_*au*_ score (IQR indicated by error bars), grouped by tumor type, as a measure of the interaction strength for each pair of proteins given in the headings. In addition, the interquartile range (IQR) and the result of the Kruskal–Wallis test are reported. Several interactions our approach identifies are well known and belong to the mTOR pathway or regulate the cell cycle or apoptosis. Some interactions are highly differential between cancer types, e.g., EGFR and HER2, have particularly strong inferred interactions in glioblastoma, head and neck squamous cell carcinomas, and lung cancer and the strongest interactions between Cyclin B1 and FOXM1 are predicted for endometrial carcinoma and ovarian cancer. The reported influence on phosphorylation of BAD at site S112 by P38-MAPK and Tuberin at site T1462 by AKT is reflected in high *LRP*_*au*_ scores. Interactions marked with “*” have been reported in the Reactome database. More predicted interactions can be found in the Supplementary materials.

In the following, we validated the strongest interactions inferred by our explainable AI approach (Fig. 1) by comparing the findings with published experimental data (indicated by ↦). The IQR and the *p* value of the Kruskal–Wallis test that compares the sample distribution between tumor groups are reported in Fig. 1 for the strongest predicted protein interactions. Since close relationships can be expected between proteins and their phosphorylated variants, we excluded them from Fig. 1 and from the following comparison to previous reported interactions.

Strong inferred interactions (high absolute undirected LRP values (*LRP*_*au*_)) were found for proteins within the mTOR pathway, e.g.,

- mTOR—Raptor: median *LRP*_*au*_ score 1.0. ↦ Reactome.

4E-BP1 showed several strong predicted interactions:

- 4E-BP1—EIF4E: median *LRP*_*au*_ score 0.74. ↦ Reactome.
- 4E-BP1—S6: median *LRP*_*au*_ scores 0.79/0.8. ↦ Regulation of 4E-BP1 and P70 S6 kinase by mTOR by phosphorylation^26^.

The interaction between 4E-BP1 and S6 was pronounced in kidney renal clear cell carcinoma (KIRC).

AKT showed particularly strong interactions in glioblastoma (GBM) and uterine corpus endometrial carcinoma (UCEC):

- AKT—GSK3: median *LRP*_*au*_ scores 0.74/0.66/0.8/0.75. ↦ Reactome.
- AKT—Tuberin: median *LRP*_*au*_ score 0.68. ↦ Reactome, AKT phosphorylates Tuberin at site T1462^27^.

Further predicted interactions were well characterized in the literature.

- GSK3—Tuberin: median *LRP*_*au*_ scores 0.83/0.81. ↦ GSK3 phosphorylates Tuberin^28^. The interaction with GSK3, but not GSK3_*αβ*_, is registered in Reactome.
- NF*κ*B—Tuberin: median *LRP*_*au*_ score 0.98. ↦ Both are phosphorylated by GSK3^28^.
- Rictor—Tuberin: median *LRP*_*au*_ score 0.68. ↦ Reactome.
- *β*-Catenin—E-cadherin: median *LRP*_*au*_ score 0.91. ↦ Reactome.
- EGFR—HER2: median *LRP*_*au*_ score 0.96. ↦ Reactome.

The LRP scores between these two proteins were highest in head and neck squamous cell carcinoma, lung adenocarcinoma (LUAD) as well as GBM. The interaction between LCK and SYK was particularly differential between tumors. It was strongest in adenoid cystic carcinoma; however, the number of patients with this cancer was the lowest in the data set (*n* = 46), possibly reducing the quality of this prediction.

- LCK—SYK: median *LRP*_*au*_ score 0.77. ↦ Reactome.
- LCK—PI3K: median *LRP*_*au*_ score 0.69. ↦ Reactome.
- EGFR—SHC: median *LRP*_*au*_ score 0.8. ↦ Reactome.
- BAD—P38-MAPK: median *LRP*_*au*_ score 0.77. ↦ P38-MAPK has been shown to regulate the phosphorylation of BAD at site S112^29^.
- MEK1—P38-MAPK: median *LRP*_*au*_ score 0.65. ↦ Reactome.

The strongest interaction (median 1.5) was found for MAPK and MEK1.

- MAPK—MEK1: median *LRP*_*au*_ score 1.5. ↦ Reactome.
- MAPK—SRC: median *LRP*_*au*_ score 0.67. ↦ Reactome. This interaction was the most homogeneous interaction in Fig. 1 across tumors.
- BCL2—BIM: median *LRP*_*au*_ score 0.79. ↦ Reactome.
- BCL2—p27: median *LRP*_*au*_ score 0.66. ↦ BCL2 upregulates p27^30^.
- Caveolin1—Collagen VI: median *LRP*_*au*_ score 0.67. ↦ Reactome.
- c-Jun—JNK: median *LRP*_*au*_ score 0.6. ↦ Reactome.

The interaction between Cyclin B1 and FOXM1 was particularly pronounced in UCEC and ovarian cancer.

- Cyclin B1—FoxM1: median *LRP*_*au*_ score 0.88. ↦ Reactome.
- MEK1—YB1: median *LRP*_*au*_ score 0.68. ↦ Interaction has been shown for acute lymphatic leukemia^31^ and colorectal cancer^32^.
- S6—YB1: median *LRP*_*au*_ scores 1.04/0.67. ↦ YB1 has been shown to be a downstream target of S6 kinases that is an essential mechanism for the survival of breast cancer cells^33^.

Our analysis yielded additional, less well-known or unknown, potentially novel interactions between the protein pairs N-Cadherin and BID, Stathmin and BID, Caspase-7 and Lck, Fibronectin and PAI-1 as well as p21. For the following predicted interactions with high LRP score, the proteins had a similar functional context:

- BID—Stathmin: median *LRP*_*au*_ score 0.72. ↦ Relevance in apoptosis^34^.
- BID—N-Cadherin: median *LRP*_*au*_ score 0.8. ↦ Regulatory role associated with the cell cycle^35^.
- Caspase-7—LCK: median *LRP*_*au*_ score 0.78. ↦ Participate in regulation of apoptosis^36^.
- Fibronectin—PAI-1: median *LRP*_*au*_ score 0.78. ↦ Both are regulated by TGF-*β*^37,38^ and their interaction might therefore be of an indirect nature.
- Fibronectin—p21: median *LRP*_*au*_ score 0.66. ↦ Fibronectin has been shown to suppress p21 expression^39^.

More predicted interactions can be found in Supplementary Figs. 1 and 2.

### Reconstruction of regulatory networks for individual patients

**Fig. 2:**
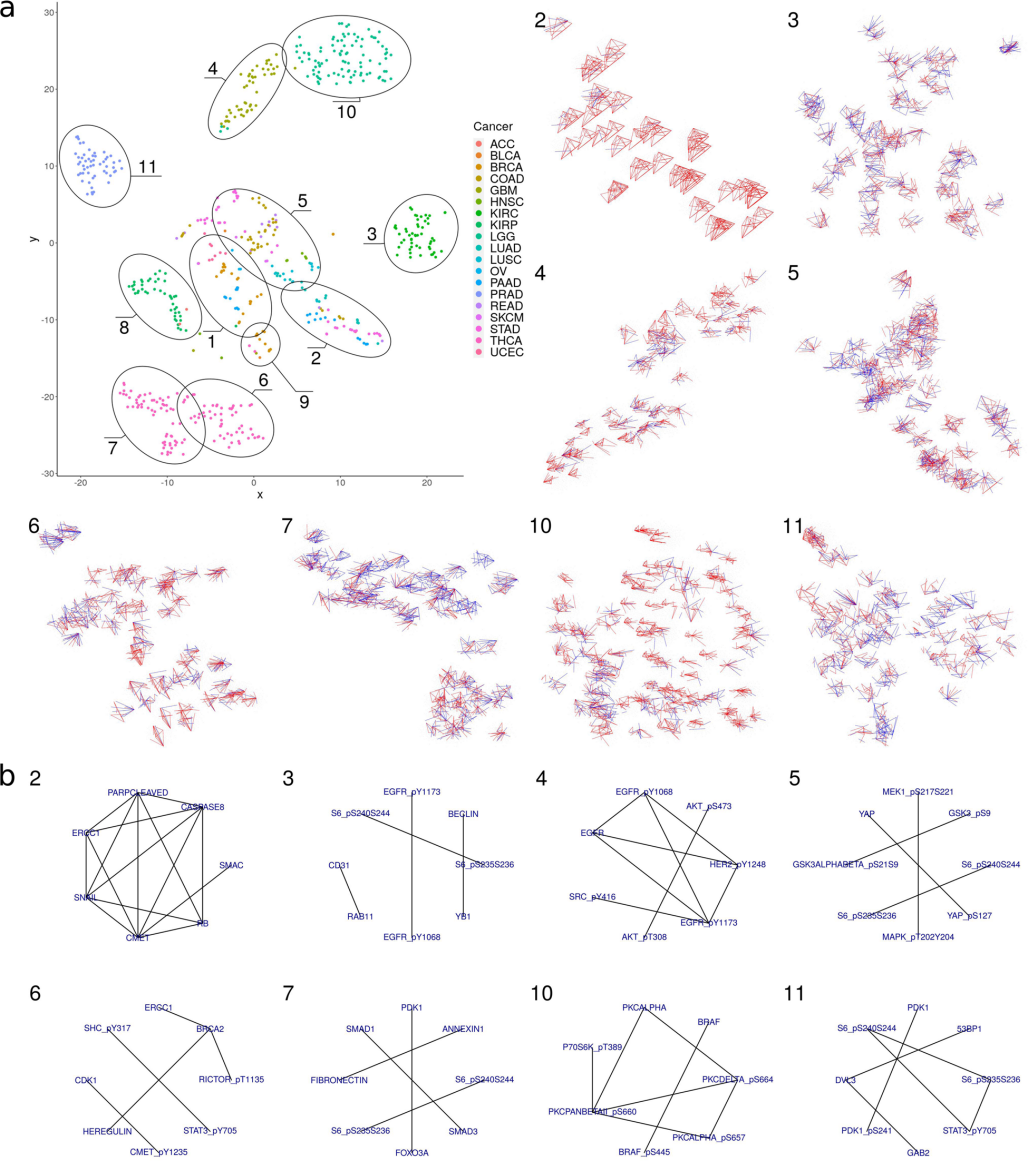
Interaction network prediction for individual patients. **a** t-SNE of tumors according to their predicted protein interactions. Every dot refers to one tumor sample. Multiple groups of samples can be separated, and in many cases, tumors of the same cancer type fall into the same cluster. Cancer types that build distinct groups are thyroid cancer (C6, C7), clear cell renal carcinoma (C3), and prostate adenocarcinoma (C11). Samples of the brain cancers glioblastoma (C4) and low-grade glioma (C10) are embedded in close proximity, but can still be separated from each other. The subplots in (**a**) illustrate the individual interaction networks for the eight largest clusters from the t-SNE analysis (additional clusters can be found in Supplementary Figs. 3 and 4). The interaction graphs are arranged according to their computed position in the t-SNE analysis to show differences in networks within one cluster. For illustration, only the strongest 0.1% of raw LRP scores are displayed (red: *LRP*_*r*_ > 0, blue: *LRP*_*r*_ < 0). Samples within the same cluster had similar strongest interactions and some clusters are tumor type specific (e.g., C3, C6, C7, C11), whereas others contain different tumor types (e.g., C2, C5). The finding that highly similar predicted network features can be found across certain tumor types is particularly clearly visible for cluster 2. **b** Median interaction network for each of the clusters in (**a**). Interactions with EGFR play an important role in cluster 4 (glioblastoma), while Beclin and YB1 are important in cluster 3 (renal clear cell carcinoma). The group of homogeneous networks in cluster 2 shows the strongest interactions between cMET, ERCC1, Caspase-8, Snail, PARP, Rb, and SMAC.

While our approach demonstrated the ability to reconstruct protein interactions averaged over samples from the same tumor type, tumors of the same entity may show substantial differences among individual patients. Therefore, for clinical diagnostics as well as for research it would be of interest to infer regulatory networks for individual tumors. In the following, we applied our approach to examine the individual interaction networks of tumors of the TCPA data set. To compare the interaction networks of individual patients we performed a t-SNE analysis (Fig. 2a) based on the predicted interaction strengths (10,731 *LRP*_*au*_ scores between every pair of proteins), which showed that patients could be separated into different groups, depending on their protein interaction networks. First, in many cases, tumors of the same cancer type were clustered together, indicating that they exhibited a similar inferred interaction profile and can therefore be assumed to be functionally similar. Prostate adenocarcinoma (C11), thyroid carcinoma (THCA, C6, C7), kidney renal papillary carcinoma (C8), and KIRC (C3) could be well separated from other cancers. The brain cancers GBM (C4) and lower-grade glioma (C10) also formed two distinct, but relatively close clusters compared to the other tumor types. While the t-SNE analysis suggests that these tumors’ inferred protein interaction networks are closely related, THCA is an example of cancer for which protein interactions were distributed among several different clusters (C6, C7, C9). Second, certain interaction network clusters were not dominated by one cancer type but composed of tumors of several types pointing to the existence of tumor type-independent proteomic network features.

A closer examination of the strongest inferred interactions (Fig. 2a) underlined that the inferred interaction patterns were mostly conserved across tumors of the same cluster, although differential regulatory patterns exist even within clusters (e.g., GBM in cluster 4 and THCA in cluster 7). Some interactions were specific for their cluster, e.g., the interaction between RAB11 and CD31 showed strong associations in cluster 3 (KIRC), while Fibronectin and Annexin-1 showed strong associations in cluster 7 (thyroid cancer).

A group of cancers that contained stomach adenocarcinoma, LUAD, pancreatic adenocarcinoma, colon adenocarcinoma, and rectal adenocarcinoma formed one cluster (C2) and showed very similar protein networks (Fig. 2a, cluster 2). The most important network features were the proteins PARP, Caspase-8, Snail, c-Met, ERCC1, and RB. Importantly, these predicted network patterns that appear to be highly conserved across these samples have also been reported in a study that examined protein regulation in a cohort of LUAD (see also discussion)^40^. Further analysis showed that the inferred interaction strength between these proteins had a bimodal distribution in cancers of the gastrointestinal tract, the lung, and the uterus while LRP scores for other tumors only were distributed around the lower peak (see Supplementary Fig. 5). These two peaks imply that certain tumor samples show concerted pathway activity not present in other tumor samples of the same type. The LRP scores between these proteins strongly correlated with Pearson’s *r* ranging between 0.7 (Parpcleaved-Snail with RB-ERCC1) and 0.99 (CMET-ERCC1 with ERCC1-Parpcleaved) between every pair of these interactions, suggesting a potential common regulatory mechanism.

### Validation of network prediction using synthetic data

**Fig. 3:**
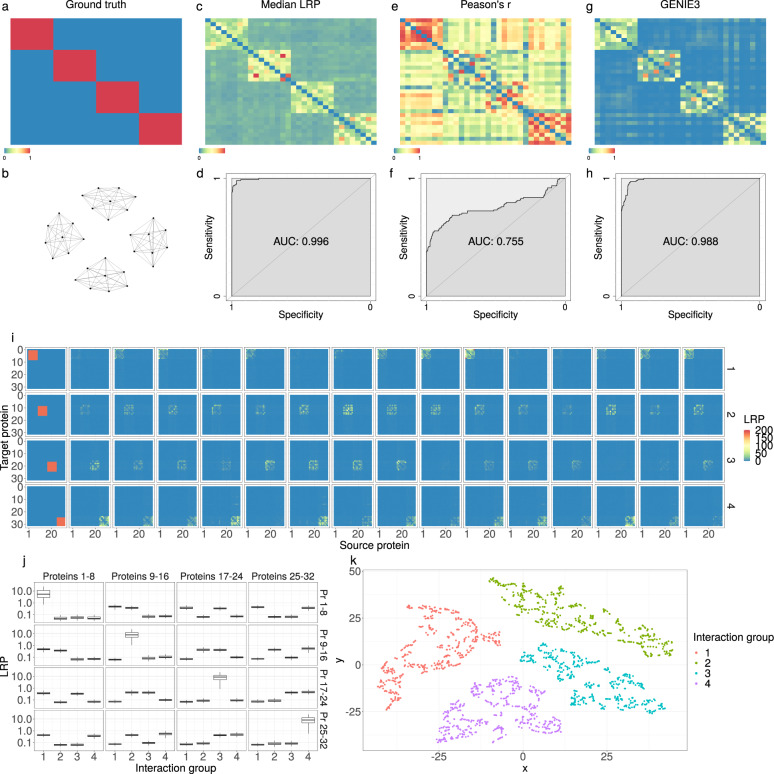
Network reconstruction on synthetic data. Synthetic data SD1: Interactions were defined only between proteins that are members of the same group of eight proteins. The existence or absence of interactions between two proteins can be represented with an interaction graph (**b**) or, alternatively, as entries of an adjacency matrix (**a**) that are set to one if two proteins interact and to zero if they do not. **c**–**h** Reconstructed interactions (top) and respective ROC analyses (bottom). *LRP*_*au*_ (**c**, **d**) and GENIE3 (**g**, **h**) can reconstruct the interaction graph almost perfectly and clearly outperform Pearson’s *r* (**e**, **f**). Synthetic data SD2: **i** Reconstruction of protein interactions for individual samples of data set SD2 with *LRP*_*au*_. In each sample, only members of one of four protein groups interacted with each other while all other proteins had no interactions. The first column illustrates a ground truth interaction network (visualized as adjacency matrix), while all further panels of the row represent the LRP reconstruction of the interaction network for individual samples from this interaction group. Overall, interactions could be reconstructed with an AUC of 0.934. **j** Each subplot shows the averaged strength of predicted interactions between the different protein groups (logarithmic scale). Median is indicated by center line, bounds of boxes indicate interquartile range, and whiskers extend to a maximum distance of 1.5 ⋅ IQR from the hinge. High values were found specifically between proteins of the same group (on the diagonal) and only if the interaction group to which the sample belonged, permitted interactions in this set of proteins. **k** t-SNE analysis of samples according to their reconstructed interactions. Each point represents one sample, and colors indicate the four different interaction groups. Samples that had the same interactions are separable from other samples.

We validated our method using synthetic data to demonstrate the capability of LRP to predict interaction networks. To this end, we created two synthetic data sets, SD1 and SD2, which each consisted of 4000 samples with 32 proteins with known synthetic interactions. SD1 contained homogeneous data in the sense that all synthetic cancer samples had the same interaction network (Fig. 3a, b). Each sample consisted of four different groups of eight proteins and interactions were set to exist only between members of the same group, but not between proteins of different groups. We compared the interactions inferred by *LRP*_*au*_ with the interactions inferred by Pearson’s correlation coefficient as well as the tree-based method GENIE3^6^ as baselines, which are common methods for the reconstruction of interaction networks from cohort data. *LRP*_*au*_ (Fig. 3c, d; AUC = 0.996, CI = 0.993–0.999) and GENIE3 (Fig. 3g, h; AUC = 0.988, CI = 0.983–0.993) clearly outperformed correlation between proteins (Fig. 3e, f; AUC = 0.755, CI = 0.709–0.800) as a measure for interaction strength.

The second synthetic data set, SD2, contained inhomogeneous data in the sense that each sample was based on one of four interaction groups. Each group allowed interactions between proteins within a set of eight proteins (Fig. 3k), while all other proteins had no interactions in this group. In each group, a different set of eight proteins was selected. This task introduced an additional level of difficulty, because the neural network had to predict the correct interaction network for each individual sample. Our LRP-based approach identified the correct interactions for individual samples with an AUC of 0.934 (CI = 0.933–0.935). The inference of interactions with one of the current state-of-the-art methods for individual network prediction, LIONESS^13^, using Pearson’s *r*, returned an AUC of 0.893 (CI = 0.892–0.894). Due to the choice of true interactions in this synthetic data set, many interactions were homogeneously missing in all samples. When we evaluated only those interactions that existed in some samples while missing in others, LRP (AUC = 0.956, CI = 0.955–0.956) outperformed lionessR even more clearly (AUC = 0.739, CI = 0.737–0.741).

## Discussion

Developments of targeted precision cancer therapies have mostly relied on understanding oncogenic mechanisms obtained from functional experimental studies of model systems. However, these approaches have limited utility in capturing the complex molecular landscape across individual patients observed in routine diagnostics samples beyond the major oncogenic drivers^41,42^. To exploit the large numbers of available diagnostic samples and to improve the mechanistic insight into oncogenic processes, we presented a method based on explainable AI capable of inferring protein interaction networks from protein expression data for single tumor samples of individual patients.

The reverse-engineering of interaction networks based on expression data has gained interest with the increasing availability of next-generation sequencing methods and several approaches^5,9^ have been proposed. Many of these methods have in common that they reconstruct an interaction network based on a set of samples and thus return an average representation over all the underlying interaction networks irrespective of their individual variability. However, due to the emergence of comprehensive molecular analysis in routine cancer diagnostics, molecular profiles are becoming increasingly complex and show substantial variability even in patients with the same cancer^43–45^. While functional measurements of patient-derived models are difficult in a routine diagnostic setting, averaged cohort analyses of FFPE tissue samples do not capture important individual patient differences. Inferring (functional) interaction networks for individual patients from (non-functional) proteomics measurements of cancer tissue samples would therefore be one prerequisite for understanding functional implications of molecular profiles and ultimately to support targeted therapy selection in a routine diagnostic setting^9^. Approaches that try to model gene pathways for individual patients ("N-of-1 methodologies”^9^) often rely on several samples from the same patient or on additional information like gene ontology^46,47^. While several methods have been developed to detect enrichment of disease genes in individual patients^9,46,47^, inferring the interaction strength between pairs of proteins (or e.g., genes) can help reveal mechanisms and reconstruct functional networks.

Unlike previous methods, our approach relies on a neural network model and explainable AI. By using the absolute value of the LRP score as a measure of functional dependence between proteins, we focus on the interaction strength between proteins. Thus, our method can pick up nonlinear relationships between proteins that may include positive and negative effects (LRP values) that would otherwise sum up to zero effect. In our validation experiments, LRP performed at least as well as GENIE3, one of the current state-of-the-art models for the prediction of average networks. However, the full potential of LRP lies in the simultaneous prediction of the underlying interaction networks for individual patients. It identified interaction networks in heterogeneous data (SD2) on a single-sample basis with high discriminatory power. This property facilitates the reconstruction of interaction networks for individual patients after training the neural network on a data set consisting of the combined data of many different cancers. Since GENIE3 only predicts average networks over samples, we compared the performance of our method for this task against LIONESS combined with Pearson’s *r*, which has been used for the analysis of biological data in the original paper^13^. LIONESS is a recent approach to predict an individual interaction network based only on expression values by reconstructing the average interaction network of a whole data set with and without a particular sample. Subsequently, the network of an individual tumor is estimated based on the difference between the two average networks. This explanation procedure, however, depends on the data distribution (e.g., if duplicate or strongly similar examples are present in the data set). Our approach outperformed LIONESS at detecting interactions for individual tumor samples, especially when focusing on the identification of interactions that are differential between samples. While LIONESS’s performance decreased for these interactions, LRP showed very stable results and the AUC even slightly increased.

To estimate the effect of a pharmacological intervention on a protein and thus a signaling path, it may be necessary to predict the causal direction of an interaction between two proteins. Since there are two LRP scores computed between every pair of proteins, future studies with larger data sets should evaluate if this can provide further information about the causal structure of interaction networks.

Similar to many other network prediction algorithms^6,8^, we report a measure for interaction strength on a continuous scale. The lack of a clear rationale to define thresholds is consistent with the fact that regulatory relationships between proteins can often not be regarded as binary (i.e., existing or not existing), but that they have variable strengths (binding kinetics) from very weak to strong.

A substantial number of the interactions predicted with our approach are validated by well-established knowledge from experimental studies, such as interactions among proteins of the mTOR pathway (mTOR, AKT, Rictor, Raptor, S6, TSC2 (Tuberin), and 4E-BP1) that received top interaction scores with our approach^48^. At the same time, most predicted interactions differed significantly between cancer types. Other predicted interactions are less well-established by previous studies and here our results may contribute to formulate novel hypotheses on so far unknown, but potentially relevant mechanisms. Whether these predicted interactions correspond to true functional relationships between these proteins, certainly requires future experimental validation.

The investigation of interaction networks revealed, in most cases, an expected strong dependence on the tumor type^49^. However, a substantial number of individual tumors of the same cancer type showed differential protein interactions, e.g., the interaction networks of some THCAs were separated into three different groups. The molecular network features of one of these groups, cluster 2, appeared to be less tumor type specific and contained, apart from thyroid cancer, tumors from the gastrointestinal tract, pancreatic cancer, and endometrial as well as cervical carcinoma. The dominant network features in tumors of this cluster were formed by the proteins c-Met, ERCC1, Caspase-8, Snail, PARP, and RB. This expands the results of Datta et al. who described this regulatory pattern for LUAD using a partial least squares method^40^. The largest regulatory network they found included ERCC1, PARP, Snail, c-Met, Caspase-8, and Rb, but connections to RB were reduced in a subgroup that showed tumor progression. While clinical information about the tumors in our data set is not sufficient for a similar analysis, the description of this regulatory network in Datta et al. is consistent with our results (Fig. 2, cluster 2). Furthermore, we observe that this particular regulatory pattern only appears in certain patients while it is not present in others with the same cancer.

RB is a well-known tumor suppression factor^50^. c-MET is associated with relapse of breast cancer^51^ and drug resistance in cancer^52^. Snail is associated with the epithelial-mesenchymal transition relevant for the ability of cancer to metastasize^53^. PARP has different functions and plays a role both in cell growth and DNA repair^54^, and PARP is associated with drug resistance in cancer^55^. It is hypothesized that Caspase-8 promotes cancer progression and resistance to therapy in some cancers^56^. Our results suggest that a common underlying regulatory mechanism exists between these proteins that may be related to drug resistance. However, this hypothesis certainly needs to be investigated in further studies.

The method proposed in this paper underlines the great potential of explainable artificial intelligence in cancer research^57–62^. While the prediction of sample-wise networks is applied to proteomic data here, it can in principle be applied to any kind of molecular profiling data. It may therefore contribute to the investigation of regulatory networks when large-scale observational data are abundant. The method may be applied to data obtained from routine diagnostic samples to study oncogenic mechanisms in individual patients and may in the future support predictive diagnostics in precision oncology.

Precision therapy strongly relies on the molecular characterization of individual patients’ tumors by molecular profiling. Since, in many cases, this does not sufficiently predict a tumor’s response to therapy, more functional information such as protein interaction networks could help improve therapy selection. In this study, we proposed a method that uses LRP to predict protein interaction networks for individual patients. On synthetic data, we showed that LRP predicts networks of individual samples with high precision. Using proteomic data across major cancers, we predicted protein interactions that showed a high agreement with current knowledge and the Reactome database. As an example, we found a highly characteristic network pattern consisting of the proteins c-MET, PARP, Caspase-8, Rb, SNAIL, and ERCC1, some of which are known to be related to drug resistance. Using our approach we could show that this pattern appears only in tumors of some but not all patients with certain cancer types. These findings suggest a great potential for explainable artificial intelligence for precision oncology.

## Methods

### Machine learning-based inference of protein interactions

We used a machine learning approach for inferring protein interactions from observed protein data. Our analysis consisted of two steps: First, a neural network was trained to maximum accuracy in order to predict held-out protein abundances from the remaining protein abundances. Then an explainable AI technique, specifically LRP, was applied to identify relevant interactions between proteins at the input and output of the network.

### Neural network

A fully-connected neural network model with ReLU activation between layers was trained on the training set to solve an imputation task in which for each sample the abundance of a number of proteins was hidden and had to be predicted given the observed proteins.

For every training sample, each protein was hidden with a probability *p*, with *p* drawn randomly and uniformly from [0.01, 0.99]. Drawing *p* from [0.01, 0.99] for every sample at every iteration during training results in a neural network capable of imputing the missing proteins from any number of known proteins. As a consequence, the number of hidden proteins followed a binomial distribution ${{{\mathcal{B}}}}(n,p)$ with parameter *n* fixed to the total number of proteins, and parameter *p* drawn randomly and uniformly from [0.01, 0.99].

To distinguish between zero-valued and missing proteins, proteins were given as input in the expanded form *ϕ*(*x*) = [*x*, 1 − *x*] where *x* denotes the protein value, and were set to *ϕ*(*x*) = [0, 0] if the protein was hidden.

The loss was computed as the mean squared error over the hidden proteins between the predicted protein value and the ground truth. The model was trained by gradient descent with a batch size of 250 and a momentum of 0.9. Learning rate (0.03), number of hidden layers (3), number of neurons per layer (10 * input dimension), and number of epochs (3600) were determined by 10-time repeated cross-validation, each time using a train-test-split of 50–50% (see Supplementary Fig. 6).

### Layer-wise relevance propagation (LRP)

Once the neural network was trained, we applied LRP^14,63^. The LRP method identifies which input variables of the neural network have contributed to a given predicted output. The method starts in the top layer by assigning *R*_out_ ← *y*_out_, where *y*_out_ denotes the predicted value for some protein. The method then redistributes *R*_out_ layer after layer, until it reaches the input layer. Let *j* and *k* be indices for the neurons of two adjacent layers, and

1 $${a}_{k}=\max \left(0,\mathop{\sum}\limits_{0,j}{a}_{j}{w}_{jk}\right)$$

be one neuron connecting these two layers. The notation ∑_0,*j*_ denotes summing over all neurons *j* in the lower layer plus a bias term *w*_0*k*_ with *a*_0_ = 1. The redistribution performed by LRP applies a propagation rule at each layer. In our work, we apply and extend the rules in^63^. In particular, we consider as a starting point the LRP-0/*ϵ*/*γ* rules given by:

2 $${R}_{j}=\mathop{\sum}\limits_{k}\frac{{a}_{j}\cdot ({w}_{jk}+\gamma {w}_{jk}^{+})}{\epsilon +{\sum }_{0,j}{a}_{j}\cdot ({w}_{jk}+\gamma {w}_{jk}^{+})}{R}_{k},$$

where ${w}_{jk}^{+}=\max (0,{w}_{jk})$. Neurons *j* are assumed to be positive and the neuron *k* in the next layer is assumed to be passed to a ReLU activation. The parameter *γ* can be set between 0 and *∞*, and can be seen as implementing a tradeoff between the robustness of the explanation and its bias. The larger the *γ*, the more robust the explanation; the smaller the *γ*, the closer it becomes to a gradient-based explanation. The parameter *ϵ* can be set between 0 and *∞* as well, and if set to a positive value, it encourages the LRP procedure to retain only the most salient elements of the explanation.

In practice, our neural network for protein prediction received real-valued inputs in the first layer and it had a top-level linear layer that produced real-valued outputs. For the more general neuron definition

3 $${a}_{k}=g\left(\mathop{\sum}\limits_{0,j}{a}_{j}{w}_{jk}\right)$$

with ${a}_{j}\in {\mathbb{R}}$ and the activation function $g:{\mathbb{R}}\to {\mathbb{R}}$ being either a ReLU function or an identity function, we can define the more general symmetrized LRP rule:

4 $$\begin{array}{ll}{R}_{j}\,\,=&\mathop{\sum}\limits_{k}\left[\frac{{a}_{j}^{+}({w}_{jk}\,+\,\gamma {w}_{jk}^{+})\,+\,{a}_{j}^{-}({w}_{jk}\,+\,\gamma {w}_{jk}^{-})}{\epsilon \,+\,{\sum }_{0,j}{a}_{j}^{+}({w}_{jk}\,+\,\gamma {w}_{jk}^{+})\,+\,{a}_{j}^{-}({w}_{jk}\,+\,\gamma {w}_{jk}^{-})}{1}_{{a}_{k} \,{ > }\,0}{R}_{k}\right.\\ &\left.+\frac{{a}_{j}^{+}({w}_{jk}\,+\,\gamma {w}_{jk}^{-})\,+\,{a}_{j}^{-}({w}_{jk}\,+\,\gamma {w}_{jk}^{+})}{-\epsilon \,+\,{\sum }_{0,j}{a}_{j}^{+}({w}_{jk}\,+\,\gamma {w}_{jk}^{-})\,+\,{a}_{j}^{-}({w}_{jk}\,+\,\gamma {w}_{jk}^{+})}{1}_{{a}_{k}\,{ < }\,0}{R}_{k}\right],\end{array}$$

where ${w}_{jk}^{+}=\max (0,{w}_{jk})$ and ${w}_{jk}^{-}=\min (0,{w}_{jk})$, and similarly for *a*_*j*_. This rule addresses the four cases of input and output (positive/positive, negative/negative, positive/negative, and negative/positive) separately, and recombines them into a single propagation rule. This rule reduces to the standard LRP-0/*ϵ*/*γ* rules when inputs and outputs are both positive.

In order to predict sample-wise protein interaction networks, we first choose a target protein that is always hidden while all other proteins are hidden with a probability of *p* = 0.5. We then let the neural network predict the target protein based on the proteins that are visible. The choice of *p* = 0.5 results in every combination of hidden proteins being equally likely. After the prediction of the target protein, our symmetrized LRP rule is applied at each layer from the output of the network to the input. Once the LRP procedure arrives at the input features, the contribution of a given (visible) input protein for the prediction of the target protein is obtained by summing over the two input neurons forming the protein expansion *ϕ*(*x*). This is repeated 100 times and the LRP scores are averaged over these 100 random imputations in order to average over different combinations of predicting (visible) proteins which results in raw LRP scores *LRP*_*r*_ between the target protein and all other proteins. We repeat this for every target protein to arrive at a full matrix connecting each protein to each other protein.

In order to derive a measure for the undirected interaction strength between two proteins, we use the average of the two absolute LRP values between two proteins and call it *LRP*_*au*_.

In previous experiments, we found that the best protein interaction matrices are obtained by setting the LRP hyperparameter *γ* = 0.01. We chose *ϵ* = 10^−5^ for numerical stability. This choice of hyperparameters transferred well qualitatively to the real-world data. Both training of the neural network and the computation of LRP values were conducted in Python/pytorch.

### Synthetic data for validation experiments

For the validation of our method, it was necessary to simulate a data-generating system in which interactions between features could be controlled. Our data generator consisted of a neural network *h* with two hidden layers that simulated interactions between certain pairs of proteins. Interactions were restricted to protein pairs by multiplying the fully-connected layers of the neural net with the adjacency matrix of a predefined interaction network. A 32-dimensional protein abundance vector *a*_0_ was initialized to **0** and was updated by the generator according to the following rule:

5 $${a}_{t}=h({a}_{t-1}+\epsilon )\quad \epsilon \sim {{{\mathcal{N}}}}({{{\boldsymbol{\mu }}}},\,{{\Sigma }}),$$

where ${{{\mathcal{N}}}}({{{\boldsymbol{\mu }}}},\,{{\Sigma }})$ describe a normal distribution with *μ* = **0** and the covariance matrix Σ chosen uniformly at random. *a*_50_, the protein abundance vector generated after 50 iterations, was taken as a sample for the data set, and the procedure was repeated until the requested amount of samples had been generated. A rectified linear unit was applied to the output of the first layer of *h* and a sigmoid function to the output of the second layer so that the neural network output would not diverge.

Our LRP method was validated on two different data sets, SD1 and SD2, consisting of a training set and a test set with 2000 samples each. In SD1 the artificial proteins had the same interactions in all samples. The adjacency matrix was chosen as the block matrix such that interactions between proteins were restricted to four different protein groups consisting of eight proteins each, while there were no interactions between proteins of different protein groups. LRP values were computed for all combinations of two proteins and for each sample and then the mean absolute undirected LRP score *LRP*_*au*_ was used as a measure for the interaction strength between every pair of proteins. Differences between the ground truth adjacency matrix and the reconstruction by LRP were analyzed with a receiver operating characteristic (ROC) curve and compared to the reconstruction of features when using the absolute Pearson’s correlation coefficient between two proteins as a measure of interaction strength. The noise *ϵ* that induced random differences between samples was correlated between features that simulated confounding dependencies between proteins that may occur at the hand of proteins that are not measured in the data set.

In SD2, proteins of each sample interacted according to one of four different interaction networks. Each interaction network allowed interactions only between proteins of one protein group. Each protein group consisted of 8 proteins and each protein was a member of one protein group.

These data simulate certain basic properties of protein regulation like nonlinear interactions and a network topology that consists of different communities^64^.

### Protein data and functional interaction network

Preprocessed protein and phosphoprotein data were obtained from TCPA for 5114 cancer samples and 258 measured proteins (Version TCGA-PANCAN19-L4.csv)^65^. Functional protein interaction data were obtained from ReactomeFI (FIsInGene_031516_with_annotations.txt)^66^. An interaction network was constructed by linking all proteins with described interaction in the ReactomeFI data using the R package igraph^67^. For phosphoproteins, additional interactions were defined with the non-phosphorylated protein as well as with all proteins interacting with the non-phosphorylated protein. To avoid isolated proteins without interactions, all proteins with less than four neighbors were excluded, resulting in a data set of 147 proteins used for the subsequent analyses. In effect, 1838 protein pairs interacted according to Reactome, and 8893 did not. Data were divided into training and test sets of equal size. While the size of the training data is relatively small as compared to other deep learning applications, the random selection of input proteins during training effectively generates many more training cases. We chose to only calculate LRP interactions for the test set, as we wanted to present a realistic use case that shows that LRP can infer networks on samples that have not previously been seen by the model. The training data were normalized to mean = 0 and standard deviation = 1. The normalization parameters from the training set were then used to normalize the test set.

### Further analysis

All subsequent analyses were conducted in the statistical programming language R^68^. Heatmaps were computed with ggplot2^69^, network visualizations were created with igraph^67^.

All statistical tests were two-sided and results were regarded as significant when *p* < 0.05. All confidence intervals were computed at the 95% confidence level.

The median of *LRP*_*au*_ scores was used to predict interactions for the validation tests and for the comparison of reconstructed protein interactions with the Reactome interactions, since it performed well and is more robust against outliers and we regard it as more suited to catch group features that are characteristic to most of the group’s samples.

### Network inference using synthetic data

The predefined interactions of the synthetic data set SD1 were inferred by taking the median of the absolute undirected *LRP*_*au*_ scores over all samples from the test set. The reconstruction of true interactions by the absolute Pearson’s *r* between proteins as well as the reconstruction by GENIE3 was used as baselines for network prediction performance. The Bioconductor version (Release (3.13)) of GENIE3^6^ was used as a baseline method for network prediction and in analogy to the symmetrical *LRP*_*au*_ scores, we computed the average of the two directed GENIE3 scores as a measure of interaction strength between two proteins. For the prediction of interaction networks for individual samples (SD2), the *LRP*_*au*_ values were compared to the respective ground truth adjacency matrices with ROC analyses on the basis of individual interactions. We compared our method with LIONESS^13^, a recent approach to infer interaction networks for individual samples. The R implementation of LIONESS was used^70^ and all default settings were adopted. To infer the interaction network for an individual sample of the test data set, lionessR was applied to the combination of this test sample with the training data set. This prevents lionessR from using information of the test data other than the test sample in question at inference time. This approach was repeated for each sample of the test set.

### Inference of averaged interactions for the TCPA data set

The median *LRP*_*au*_ score was used as a measure of interaction strength to receive robustness against particularly strong interactions. For 147 proteins 10731 *LRP*_*au*_ interaction scores were predicted.

A hypergeometric test (R package “stats”) was applied to test if the 100 strongest inferred interactions were more likely to be reported in the Reactome database^24^. This result was compared against the symmetrized GENIE3 scores as a baseline. The 36 strongest inferred interactions were compared to reported interactions from the scientific literature and visualized, separated by tumor type, as barplot. The Kruskal–Wallis test was applied to test if the interactions were differential between tumor types. The *p* value was adjusted for the 36 examined interactions using the Holm–Bonferroni correction.

### Comparison of individual LRP networks

In this section, protein interaction networks for individual tumor samples are compared. To allow for better visibility between individual interaction networks, a subset of 639 samples (25%) for which the neural network’s imputation results correlated best with the ground truth expression data was displayed. Furthermore, the display of raw *LRP*_*r*_ scores (instead of *LRP*_*au*_) in different colors (blue: negative *LRP*_*r*_, red: positive *LRP*_*r*_) allows for a better illustration of differences between individual networks. A t-SNE analysis was applied to compute a 2D-embedding in order to visualize similarities and differences between samples^71^. The *LRP*_*au*_ scores of each sample (10,731 LRP_*au*_ scores per sample) were used as input.

The individual interaction networks were visualized depending on their position in the t-SNE plot to show gradual differences and dependencies on the tumor type.

In order to receive an example plot for every cluster, the median *LRP*_*au*_ scores of every interaction over all samples of the cluster were taken and the strongest interactions (up to a protein count of 8) were shown as a labeled network graph.

### Reporting summary

Further information on research design is available in the Nature Research Reporting Summary linked to this article.

## Supplementary information

Supplementary Information

Reporting summary

## Data Availability

All data used in this article are available at https://github.com/PhGK/ProteinNetworkLRP10.5281/zenodo.6370802.

## Code availability

All computer code used in this article is available at https://github.com/PhGK/ProteinNetworkLRP.
