# Supplementary material for: Patient-level proteomic network prediction by explainable artificial intelligence
Source: NPJ Precis Oncol. 2022 Jun 7;6:35. doi: 10.1038/s41698-022-00278-4 (PMC9174200; doi:10.1038/s41698-022-00278-4)
Supplement: Supplementary file 1 — Supplementary Information [file 41698_2022_278_MOESM1_ESM.pdf]

Supplementary information for "Patient-level proteomic network prediction by explainable artificial intelligence"

Philipp Keyl[†], Michael Bockmayr[†], Daniel Heim, Gabriel Dernbach, Grégoire Montavon[*], Klaus-Robert Müller[*], and Frederick Klauschen[*]

[†]These authors contributed equally to this work.
[*]Corresponding authors. E-mail: G. M. gregoire.montavon@tu-berlin.de, K-R.M. klaus-robert.mueller@tu-berlin.de and F. K. f.klauschen@lmu.de.

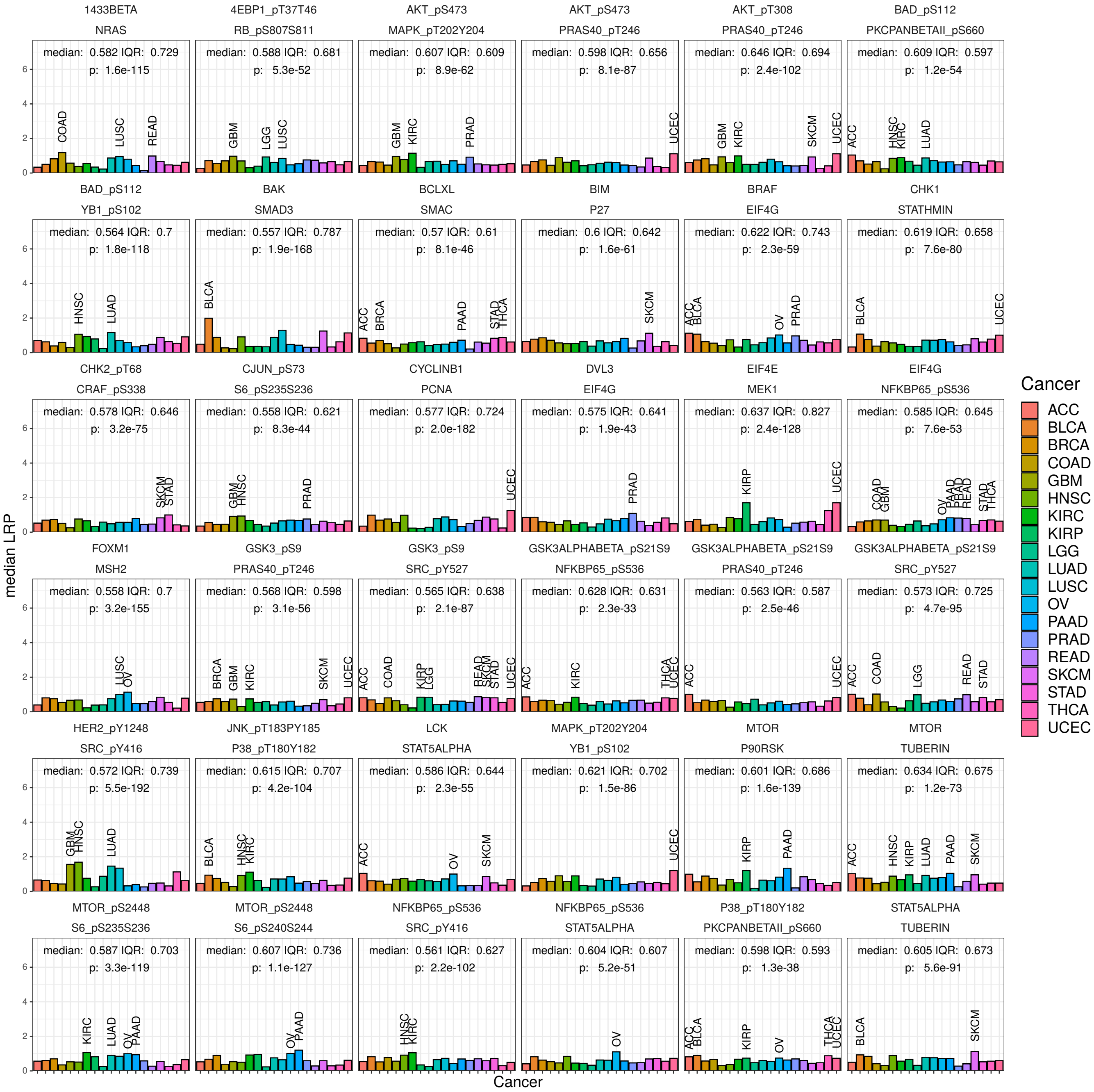

Supplementary Figure 1: Strongest inferred interactions for the TCPA data set: 37-72. Supplementary data for Figure 1 of the main manuscript.

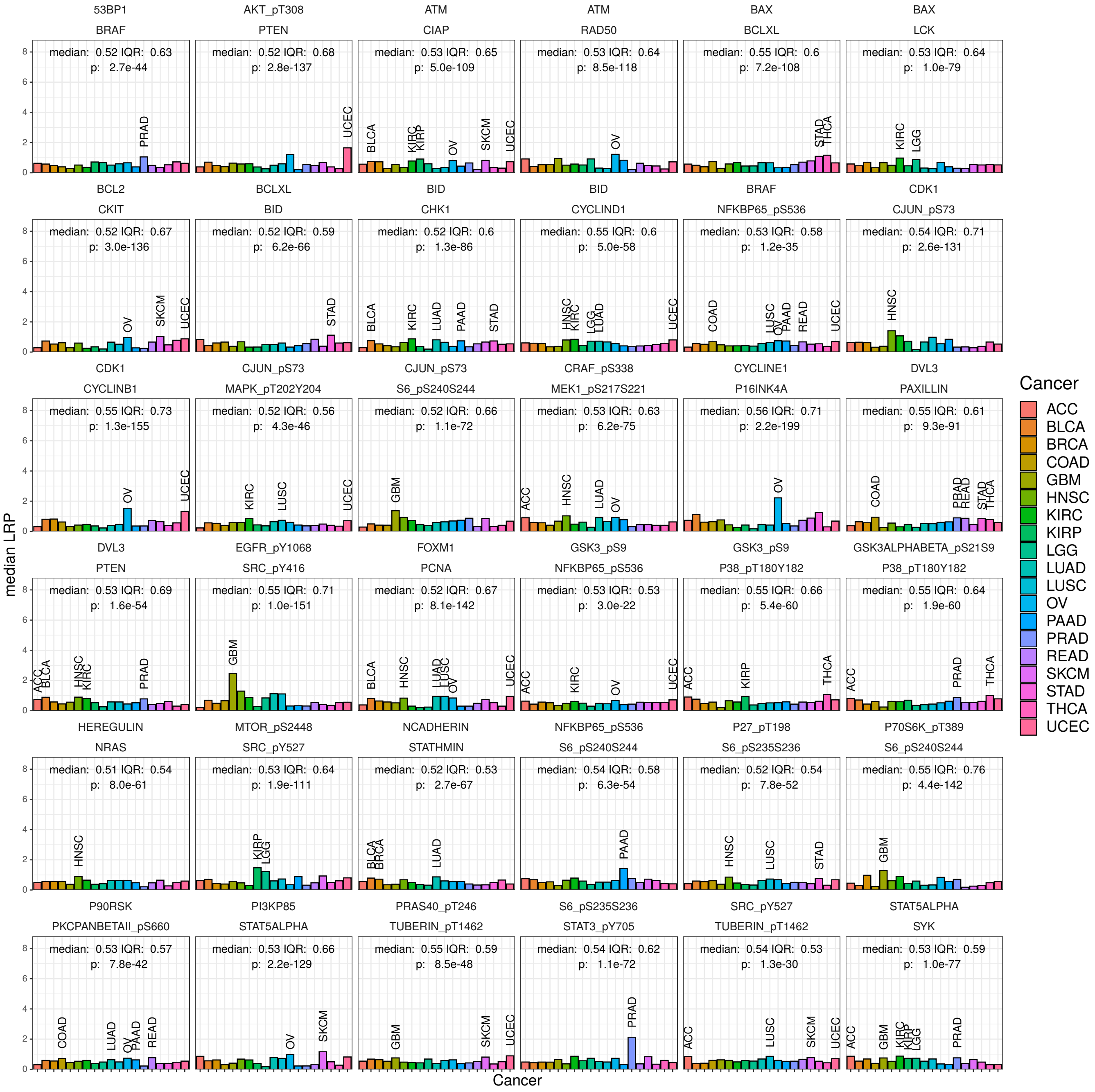

Supplementary Figure 2: Strongest inferred interactions for the TCPA data set: 73-108. Supplementary data for Figure 1 of the main manuscript.

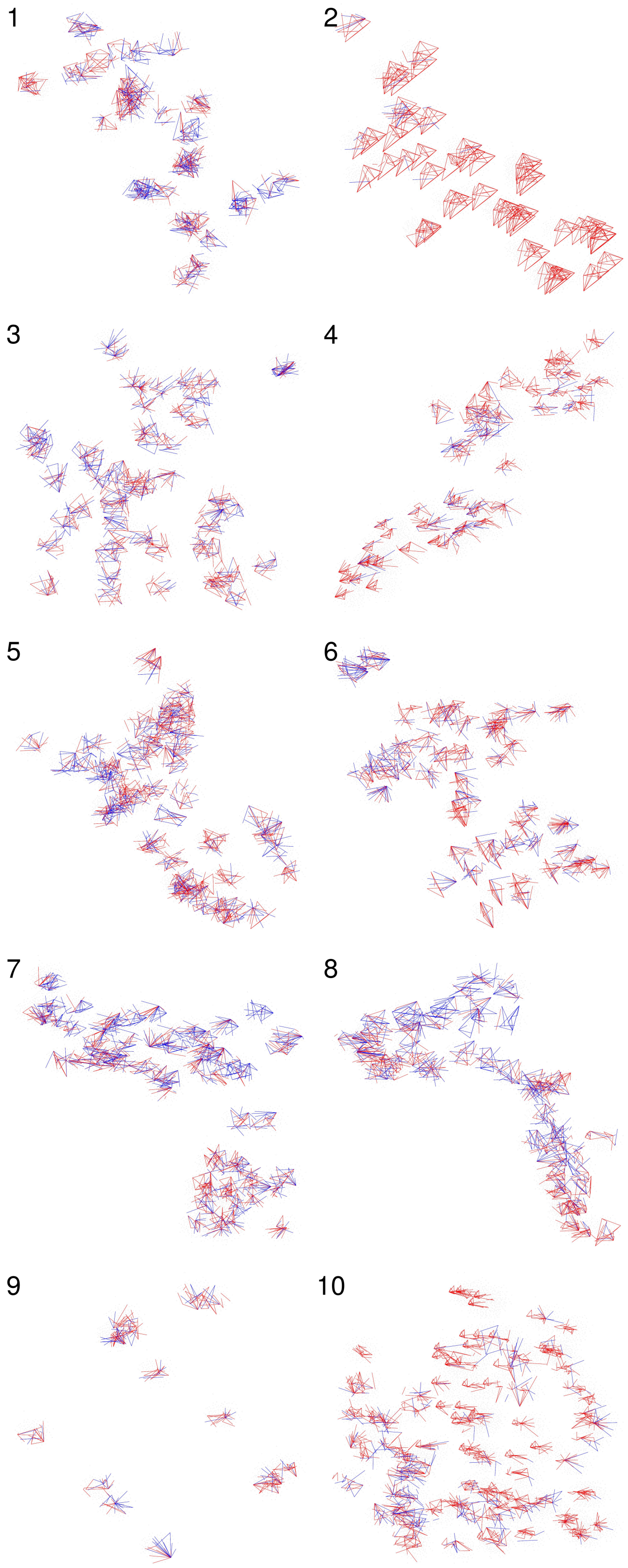

Supplementary Figure 3: Protein interaction networks for individual patients. Supplementary data for Figure 2A of the main manuscript.

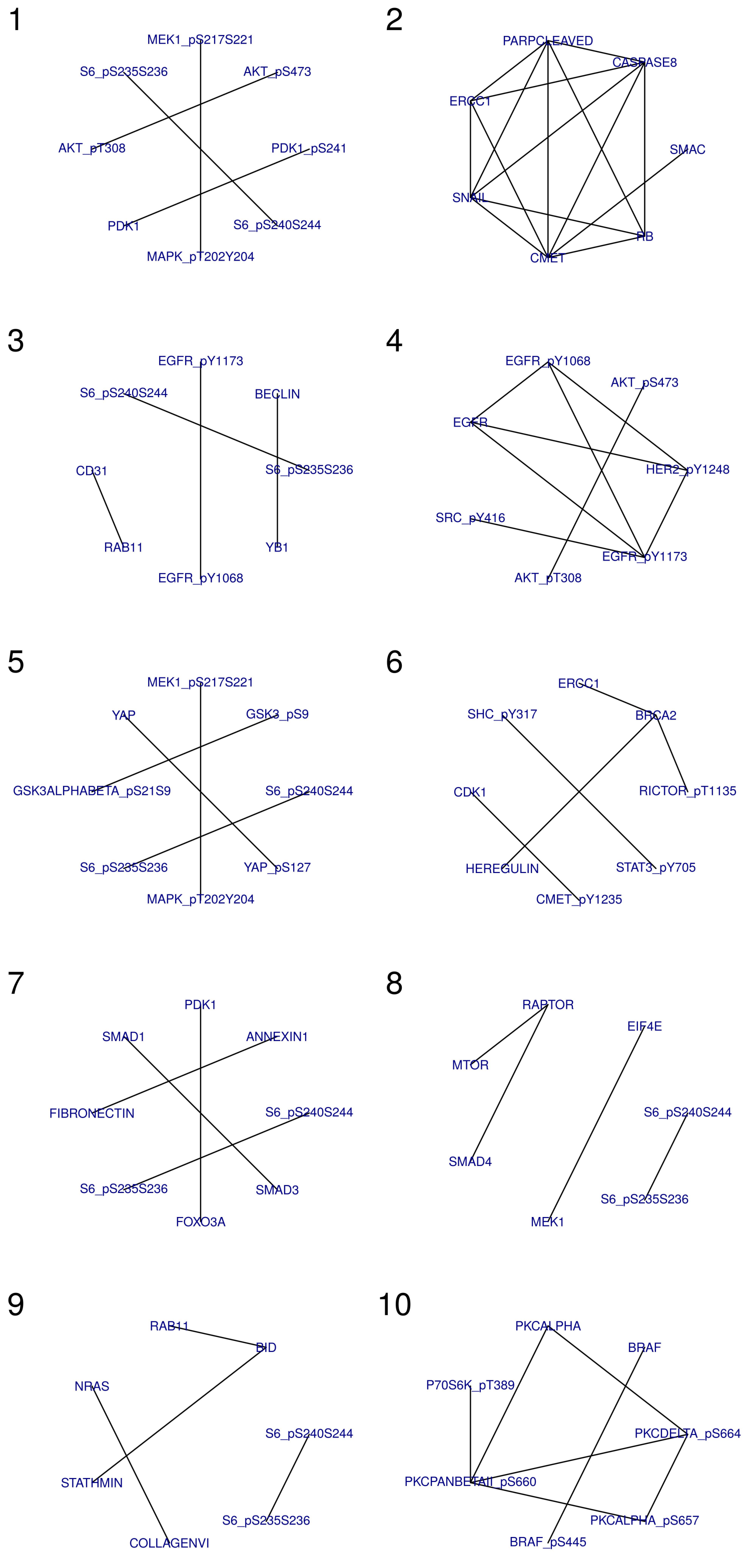

Supplementary Figure 4: Averaged interaction networks for clusters of Figure S3. Supplementary data for Figure 2B of the main manuscript.

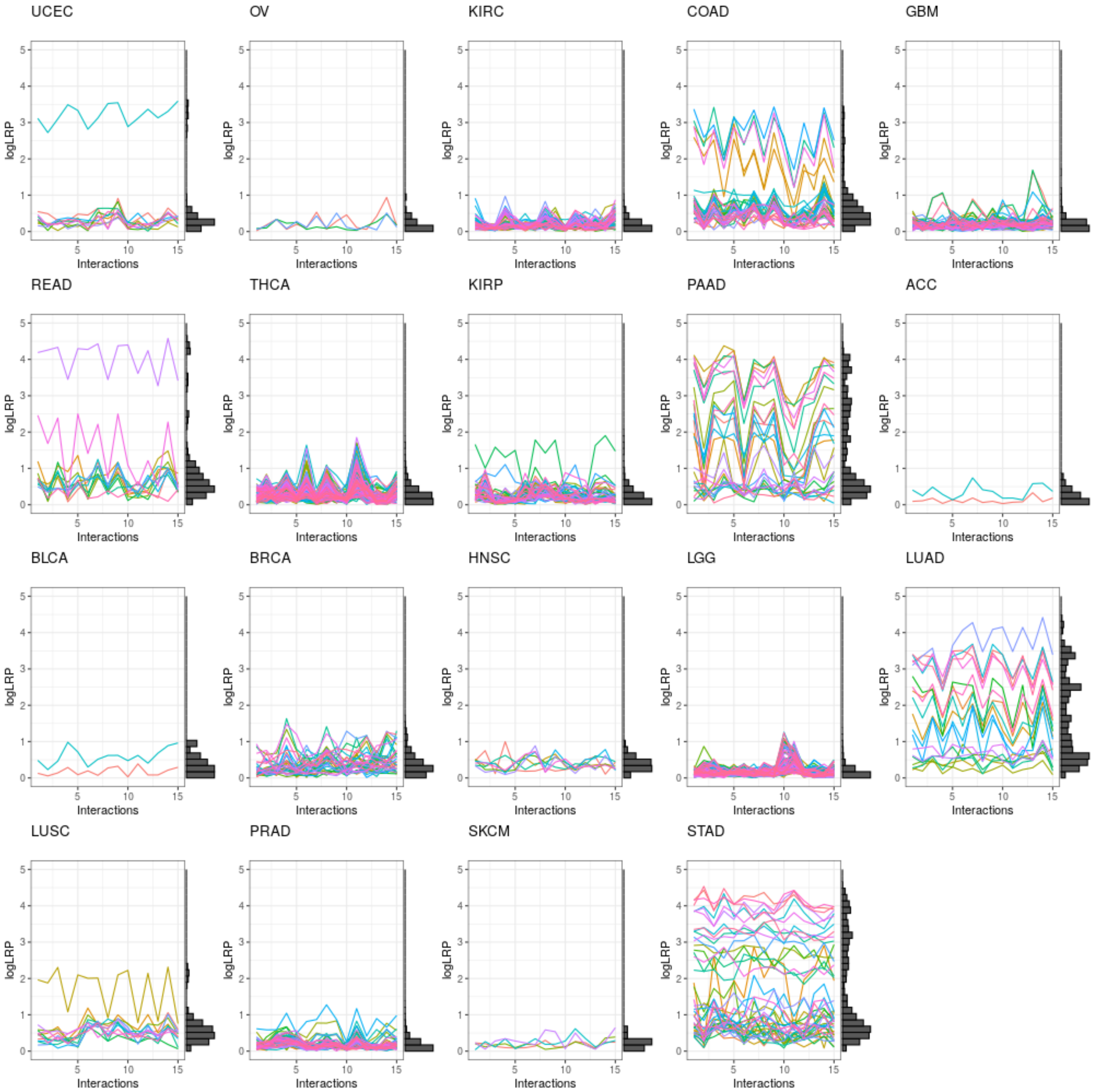

Supplementary Figure 5: Analysis of the 15 different interactions (x-axis) existing between the proteins c-MET, Snail, Caspase-8, ERCC-1, PARPcleaved and RB for the data subset used in Figure 2. The vertical axis displays the logarithm of the LRP_{au} score. LRP_{au} scores that belong to one patient are connected by a colored line. Each subplot relates to one cancer type. The distribution of LRP_{au} scores is displayed on the right side of each subplot. While in some cancers, LRP_{au} scores are overall low between these proteins, for the cancers UCEC, PAAD, LUAD, LUSC, COAD, STAD and READ interactions between these proteins show a bimodal pattern. In many cases, all LRP_{au} scores are either high or low, which could suggest that these proteins are regulated by a common mechanism.

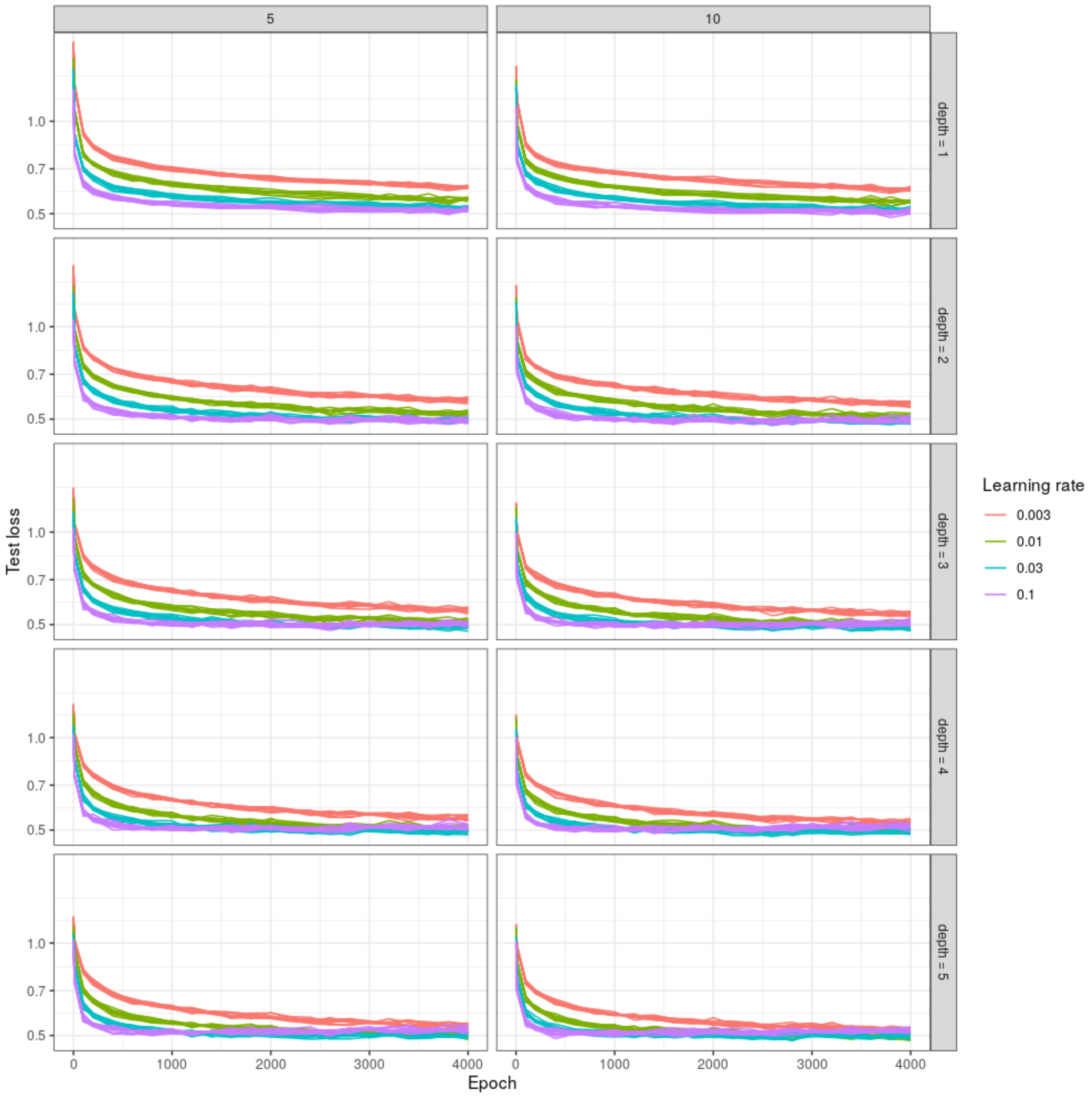

Supplementary Figure 6: Crossvalidation for model selection. We tested different choices for 'hidden layers' (rows: 1-5), 'number of neurons per layer' (columns: [5,10] x number of proteins), 'learning rate ([0.003, 0.01, 0.03, 0.1])' and 'number of epochs' (up to 4000). Every combination of hyperparameters was tested 10 times.
